# Sensitivity vs Competing Proton Transfer Reactions: Addressing Key Parameters of Ion Chemistry in Ion Mobility Spectrometry

**DOI:** 10.1021/jasms.5c00161

**Published:** 2025-08-01

**Authors:** Christoph Schaefer, Stefan Zimmermann

**Affiliations:** 529942 Leibniz University Hannover, Institute of Electrical Engineering and Measurement Technology, Department of Sensors and Measurement Technology, Appelstr. 9A, 30167 Hannover, Germany

## Abstract

Ion mobility spectrometers (IMS) are widely used in various gas sensing applications due to their high sensitivity and rapid analysis times. However, in complex gas mixtures, reactions between the protonated target analyte and interfering species can lead to discrimination of analytes with low gas basicity, reducing sensitivity or even making detection impossible. Operating IMS at low pressure and high reduced electric field strengths has been shown to mitigate these competing ion–molecule reactions. Therefore, in this work, we present a kinetic model to evaluate the effect of key operating parameters on the ion suppression caused by competing ion–molecule reactions, guiding the instrumental design of IMS. The results demonstrate that measures to reduce competing ion–molecule reactions, such as lowering the operating pressure or reaction time, also reduce sensitivity due to fewer ion–neutral collisions. However, in scenarios with high concentrations of interferents, the reduced effect of competing ion–molecule reactions is critical for detecting target analytes with low gas basicity, thereby enhancing sensitivity under such conditions. Based on these findings, decreasing operating pressure and reaction time or increasing reduced electric field strength are the most promising strategies to minimize competing reactions and, in complex chemical backgrounds, increase sensitivity.

## Graphical abstract

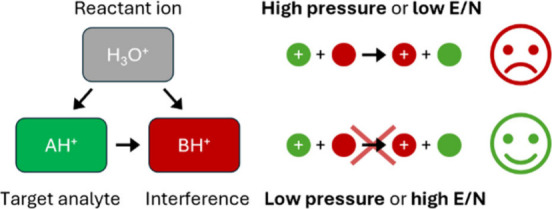

## Introduction

Ion mobility spectrometers (IMS) analyze ions based on their ion mobility in a neutral gas under the influence of an electric field. Their short analysis times of a few seconds^1^ and the potential for miniaturization render them useful for mobile applications in gas sensing.^2–4^ Consequently, IMS are employed in a wide range of applications, such as detecting explosives,^5–7^ chemical warfare agents,^8–11^ drugs,^12–14^ toxic industrial chemicals^15,16^ and other environmental pollutants.^17,18^

For detection of gaseous analytes, IMS often uses chemical ionization of the analytes through different ion–molecule reactions with reactant ions, initially formed in an ion source. Using such ionization techniques, IMS operating at ambient pressure can reach extremely low limits of detection down to the single-digit ppt_V_ (parts-per-trillion by volume) range in one second due to the high number of occurring ion–neutral collisions.^1^ Under such conditions, the most relevant reactant ions are hydrated hydronium ions H_3_O^+^(H_2_O)_*n*_, which ionize the analytes through proton transfer, forming the protonated analyte monomer.^19^ However, after this initial proton transfer by the reactant ions, the protonated target analytes can further react with interfering neutral species with a higher gas basicity (GB) present in the reaction region through competing proton transfer, forming the protonated interference monomer.^15,20–22^ This competing reaction leads to a charge loss of the target analyte, making its detection by IMS difficult, if not impossible.

Considering these effects, the reaction system in chemical ionization sources can be operated in two different regimes: kinetic control or thermodynamic control.^23–26^ Note that these operating regimes are not limited to IMS but also apply to mass spectrometry using chemical ionization sources. For a limited number of ion–neutral collisions (e.g., at short reaction times or low neutral particle densities), the amount of formed product ions for each species is accurately described by the reaction kinetics, primarily involving the initial proton transfer by the reactant ions.^21,24,27,28^ Under such conditions, the absolute number of protonated analyte monomers consumed by competing proton transfer is small–owing to both the short reaction times and the low initial number of available protonated analyte monomers. Hence, competing proton transfer has a negligible effect on the absolute ion concentrations and thus signals in IMS under these conditions. Hence, the reaction system is under kinetic control. For longer reaction times, significant amounts of protonated analyte monomer form and competing proton transfer can strongly impact the generated ion population. The reaction system then approaches an equilibrium where the neutral species with the highest GB is the most available ion species.^21,24,27,28^ Since the thermodynamic properties of the analytes (specifically, GB) determine the ion population under such conditions, the reaction system is under thermodynamic control. In an IMS operated at ambient pressure, the reaction system is typically under thermodynamic control due to the high number of ion–neutral collisions. While this can lead to the desired low limits of detection, it can also result in a limited number of ions of analytes with low GB, causing false-negative alarms.^15,20–22^ The ionization of analytes with low GB is further complicated by the formation of water clusters of reactant ions, that are formed particularly at ambient pressure.^29–31^ Since the larger neutral water clusters have a higher GB,^32^ the equilibrium of the initial proton transfer by the reactant ions can shift to the reactant ions, meaning that ionization especially of analytes with low GB becomes difficult if not impossible.^30,33,34^ If the target analyte has a high GB, the competing proton transfer reactions can be beneficial, as they can suppress spectral interfering compounds. Therefore, operation at ambient pressure maximizes the sensitivity for these analytes, as further competing reactions between analytes and interferents are unlikely. However, in many applications, the target analyte might not have the highest GB, especially when multiple target analytes need to be detected in complex samples.

One approach to address this issue is coupling IMS with a preceding separation dimension, such as gas chromatography (GC).^35–38^ If the gaseous analytes are sufficiently separated before being introduced to the IMS, the negative effects of competing ion–molecule reactions can be mitigated, resolving false-negative alarms. However, this approach significantly increases analysis times to several minutes, compromising the rapid analysis of IMS. Another strategy for reducing the effect of competing proton transfer is to reduce the operating pressure, thereby decreasing the number of ion–neutral collisions. For instance, the High Kinetic Energy Ion Mobility Spectrometer (HiKE-IMS) operates at 10 to 40 mbar,^39^ leading to kinetic control of the ion population.^40,41^ The low neutral particle density at these operating pressures also allows for high reduced electric field strengths *E*/*N*, leading to short reaction times and further allowing for kinetic control. Operation at high *E*/*N* also results in dissociation of water clusters of reactant ions, ensuring ionization via the bare reactant ions, which is particularly beneficial to efficiently ionize and thus detect analytes with low GB by proton transfer.^33,42^ However, the lower number of ion–neutral collisions results in lower reaction rates for initial proton transfer by the reactant ions, causing higher limits of detection.^15^ Hence, since both the reaction rates of initial and competing proton transfer decrease at these low operating pressures, a compromise for detecting analytes with low GB needs to be found that ensures sufficient analyte ionization while still reducing proton transfer reactions between analytes and interferents. Consequently, current research focuses on increasing the operating pressure of HiKE-IMS. In this context, previous work from our group has shown that increasing the operating pressure of HiKE-IMS to 60 mbar improves sensitivity while still achieving high reduced field strengths.^43^ These correlations highlight the conflict between sensitivity and competing proton transfer reactions in IMS using chemical ionization. Either kinetic control reduces the effect of chemical cross-sensitivities caused by competing reactions at the cost of higher limits of detection or thermodynamic control results in high sensitivity (at least for analytes with high GB), but potential competing reactions can impair the detection of certain analytes in the presence of other analytes and interferents. However, operation in the transition range between kinetic and thermodynamic control is also conceivable, as indicated by the example of HiKE-IMS at 60 mbar.^43^ Choosing appropriate operating conditions can then provide a compromise between achieving both high sensitivity and reduced proton transfer reactions between analytes and interferents. Identifying such relevant operating regimes for IMS allows for tailored operation with respect to the application, such as monitoring a target analyte in a fairly “clean” background or in general a target analyte with high GB versus detecting and quantifying analytes in complex chemical backgrounds.

This work focuses on modeling the ion population in IMS under different operating conditions and regimes of the reaction system to highlight key parameters that influence initial proton transfer and thus sensitivity as well as competing reactions. By employing a kinetic model to describe ion–molecule reactions, we aim to provide qualitative insights that can guide the design of an IMS with these considerations in mind.

Several models have been used to calculate ion populations in IMS^20,21,31,42,44–47^ and selected ion flow tube mass spectrometry (SIFT-MS).^48,49^ These models often focus on other aspects such as reactant ion formation,^45,47^ cluster formation of ions,^31,44–47^ or the formation of the product ions from a single analyte species.^42,48,49^ However, some models, like those developed by Lattouf et al. and Puton et al., consider competing ion–molecule reactions between target analytes and interfering species at ambient pressure, focusing primarily on concentration effects.^20,21^ In contrast, this work emphasizes the influence of *E*/*N* and operating pressure on competing reactions to guide the instrumental design. *E*/*N* affects reaction time while operating pressure influences the number density of gas molecules, which impact both the number of ion–neutral collisions and subsequently sensitivity and proton transfer reactions between analytes and interferents.

## Experimental Section

### Kinetic Model

The kinetic model developed in this work is designed to model the ion population in IMS in a gas mixture with several neutrals, focusing only on one single target analyte and one single interfering species in the following. It builds upon the kinetic model of Allers et al., used for modeling the reactant ion population and product ion formation in HiKE-IMS depending on *E*/*N*.^47,50^ While the model of Allers et al. considers the complete reaction system of reactant ion formation in air or nitrogen, the kinetic model developed in this work considers a different scheme, as depicted in Figure 1: The model assumes initial ionization of the neutral analyte species A (here: acetone, ACE) through proton transfer with H_3_O^+^. Subsequently, competing proton transfer between the protonated analyte monomers AH^+^ and the interfering species B (here: dimethylformamide and DMF) is possible. Additionally, interfering species B can also be ionized by the initial proton transfer with H_3_O^+^. The possible reactions 1, 2, and 3 of initial proton transfer by the reactant ions and competing proton transfer constitute the reaction system. Such proton transfer reactions typically proceed at the collisional rate, if the reaction is exothermic by more than 25 kJ/mol.^51^ Since in most cases the analyte concentration ranges from ppt_V_ to low ppm_V_, it is unlikely that the reactant ions undergo three-body collisions with both neutral molecules A and B but instead undergo single collisions with either A or B. Hence, the presence of the interferent B does not prevent the initial formation of the target analyte ions in reaction 1 but rather leads to their consumption with reaction 3.

$$H_{3}O^{+}+A\to AH^{+}+H_{2}O$$ 1

$$H_{3}O^{+}+B\to BH^{+}+H_{2}O$$ 2

$$AH^{+}+B\to BH^{+}+A$$ 3

**Figure 1:**
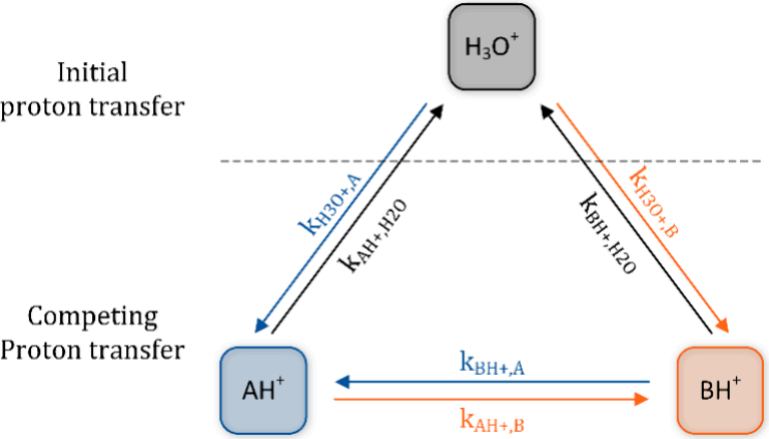
Basic operating principle of the kinetic model assuming initial proton transfer from H_3_O^+^ to two exemplary analytes A and B and competing proton transfer between both analytes.

Studies on ion–molecule reactions have shown that the hydration of reactant ions can have a substantial impact as it can affect the reaction kinetics^48^ or even completely prevent analyte ionization.^30,34,52^ However, including the effect of ion–solvent clustering is not within the scope of this work, so we include only the reactions of H_3_O^+^. This assumption seems especially reasonable at high effective temperatures, where such ion–solvent clusters dissociate.^47,53^ In any case, the modeling results present a best-case scenario for analytes whose ionization is not strongly inhibited by the hydration of reactant ions. For such analytes, we expect a reasonable agreement for modeled data with actual experiments. Since the hydration of H_3_O^+^ only shows a small effect on the ionization of acetone but does not completely prevent its ionization up to the cluster H_3_O^+^(H_2_O)_3_,^54^ acetone is considered as a reasonable target analyte. Note that such ion–solvent clusters are not only crucial for chemical ionization sources but also for electrospray ionization, where removing large, charged droplets is of importance.^55,56^

The change in the concentration of AH^+^ can be described by the differential equation in eq 4, involving the reaction rate coefficients *k*_for_ of the reactions 1, 2, and 3, as well as the concentrations of the participating neutral and ion species.

$$\frac{d\left[AH^{+}\right]}{dt}=k_{\mathrm{for},1}\left[A\right]\left[H_{3}O^{+}\right]-k_{\mathrm{for},3}\left[B\right]\left[AH^{+}\right]$$ 4

To calculate the ion population over time, a system of such differential equations describing the concentration changes of H_3_O^+^, AH^+^, and BH^+^ is established, including reactions 1 through 3. An in-depth explanation of all calculations within the kinetic model, including the calculation of reaction rate coefficients, can be found in section S1 in the Supporting Information. Briefly, the reaction rate for proton transfer from the species with lower GB to the species with higher GB is assumed to proceed at the collisional rate, which is calculated using the parametrization from Su and Chesnavich.^57^ This is common practice in SIFT-MS and PTR-MS,^58,59^ that both rely on the same ion–molecule reactions as IMS, assuming that proton transfer reactions occur at each collision if the reaction is exothermic by more than 25 kJ/mol.^51^ All reaction rate coefficients of the initial and competing proton transfer for different *E*/*N* values as well as the corresponding effective temperature of the proton-donating species can be found in the Supporting Information. Additional molecular properties relevant to the model are summarized in Table 1. Note that the collisions between the ions and neutral molecules in IMS are characterized by the effective temperature *T*_eff_ according to the Wannier equation.^60,61^ When elevated reduced field strengths are considered, this effective temperature is used to calculate the collision rate coefficients instead of the absolute temperature.

**Table 1:** Molecular Properties of Analytes Relevant for the Modeled Ion Population and Reduced Ion Mobilities in the Low-Field Limit of Their Protonated Monomers H_3_O^+^, ACE·H^+^, and DMF·H^+^ ^a^

Substance	Molecular mass (Da)	Dipole moment (D)	Polarizability (Å^3^)	Low-field ion mobility (cm^2^/(V s))	Gas basicity (kJ/mol)
Water	18.02	1.857 ^70^	1.501 ^71^	2.7489	660.0 ^72^
ACE	58.08	2.880 ^73^	6.270 ^74^	2.2440	782.1 ^72^
DMF	73.09	3.820 ^73^	7.809 ^74^	2.0758	856.6 ^72^

a The ion mobilities are calculated with MobCal-MPI.^62^ Note that the dipole moment and polarizability of the water molecule are included for completeness but are not required for calculating the reaction rate coefficients of reactions 1 and 2.

This work assumes an IMS configuration that uses a time-independent, homogeneous electric field within the reaction region and a constant generation of H_3_O^+^ ions. These reactant ions are transported through the reaction region by the applied electric field, where they ionize neutral analyte molecules A and B. Consequently, the ion population significantly depends on the position within the reaction region. It is essential to note that IMS analyzes the ion population at the end of the reaction region. The reaction time *t*_R_ in such configuration is determined by the reaction region length *L*_RR_, the reduced ion mobility *K*_0_ of the reactant ions, the Loschmidt constant *N*_0_ and the reduced field strength *E*/*N* according to eq 5.

$$t_{R}=\frac{L_{\mathrm{RR}}}{K_{0}\cdot N_{0}\cdot \frac{E}{N}}$$ 5

In this work, the ion population will either be modeled depending on reaction time or position within the reaction region. Position-dependent modeling is crucial to evaluate the effects of pressure and *E*/*N* on the detected ion population, which is assumed equivalent to the ion population at the end of the reaction region. This approach involves converting the time-dependent differential equations of ion concentrations into position-dependent differential equations by dividing the time-dependent differential equations by the drift velocity of the proton-donating ion species, as demonstrated by Spesyvyi et al.^49^ Calculating the drift velocity requires the ion mobility, which is calculated for different reduced field strength using MobCal-MPI^62^ using the routine explained below. The low-field ion mobilities are summarized in Table 1. A discussion of the calculated values and its comparison to experimental data,^63,64^ if available, and to a simple estimation of the ion mobility from the ion mass as implemented in the statistical diffusion simulation (SDS) user program for SIMION^65^ can be found in the Supporting Information. For each set of parameters, the ion population depending on time or position in the reaction region is obtained by numerically solving the set of differential equations. The differential equations are solved using the MATLAB function “ode15s”, that uses numerical differentiation formulas, that are linear multistep methods of order 1 through 5.

To model the ion mobilities of all relevant ion species, we first used the ORCA program package^66,67^ to use density functional theory, applying the ωB97X-D3/def2-TZVPP level of theory to obtain the structure of the involved species. We then used MobCal-MPI^62^ to determine the low-field ion mobilities of all ion species. The field-dependence of ion mobility can have a significant impact on the ion mobilities of the considered ion species due to its effect on ion–solvent clustering, the dependence of the ion–neutral interaction potential on relative velocity, and the hard-sphere effect at high effective temperatures. Since we do not consider hydration of the ions in this kinetic model, which can have a major contribution on the field-dependence of ion mobility, we use only the low-field ion mobility values from MobCal-MPI in this work for simplicity. Detailed experimental and theoretical studies on the field-dependence of ion mobility can be found in various publications.^44,63,68,69^

For simplicity, the model relies on several assumptions. It does not account for ion loss due to neutralization at electrode surfaces or diffusion nor does it consider ion–ion recombination. In ion sources for chemical ionization, both charge types can initially be generated so that their recombination would introduce an additional loss term in the differential equations that are solved in the model, being particularly relevant when using a field-free reaction region.^75^ However, with an applied electric field in the reaction region, as considered in this work, positive and negative ions are spatially separated due to their opposing motion in the electric field, and hence, neglecting such effects seems reasonable in this kinetic model. The reactions are assumed to proceed with a constant reaction rate coefficient for a given effective temperature, neglecting effects resulting from the hydration of reactant ions or protonated monomers. Additionally, no formation of proton-bound dimers from association between the protonated monomers and other neutral analyte molecules is considered, which is a reasonable assumption when using low analyte vapor concentrations. The vapor concentrations are assumed to remain constant in the reaction region and are not affected by the reaction. This is typically valid since the ion concentration is often low compared to the neutral vapor concentration,^29^ and the continuous introduction of sample gas to the reaction region ensures constant reaction conditions. Furthermore, the field-dependence of ion mobility is neglected. These assumptions may lead to quantitative deviations between the modeled ion population and actual ion population. However, the aim of this work is to provide insights into the effects of key operating parameters on sensitivity and proton transfer reactions between analytes and interferents over a wide range. Therefore, the qualitative conclusions drawn from the results and observed trends are believed to be reasonably accurate and offer valuable guidance in the design of an IMS.

Besides the ion mobility of all ion species, the kinetic model uses *E*/*N*, temperature, pressure, reaction region length, and vapor concentrations of the target analyte and interfering species as input parameters. Table 2 summarizes the standard parameters used for all calculations, unless otherwise specified.

**Table 2:** Typical Simulation Parameters^a^

parameter	standard value
reaction region length	50 mm
reduced electric field strength	1.2 Td
operating pressure	1000 mbar
operating temperature	300 K
water concentration	70 ppm_V_

a If not stated otherwise, the typical values are used for modeling the ion population.

## Results and Discussion

In the following, the developed kinetic model will be applied to model the ion population depending on key operating parameters, such as reaction time, pressure, reaction region length, and reduced field strength. The insights will help guide the instrumental design of an IMS with respect to sensitivity and proton transfer reactions between analytes and interferents. The focus will be on ion suppression of an exemplary target analyte (acetone (ACE), GB = 782.1 kJ/mol^72^) in the presence of an exemplary interfering species with a higher GB (dimethylformamide (DMF), GB = 856.6 kJ/mol^72^). DMF is chosen because it is a common solvent with a high GB, whereas ACE is a typical model analyte for IMS. Furthermore, as described above, its ionization is only slightly affected by hydration of H_3_O^+^,^54^ making it a reasonable target analyte for this model that does not include the hydration of reactant ions.

It is obvious that the reaction time significantly affects ion population using chemical ionization,^21,24,28^ as it influences both the amount of reaction product from initial proton transfer by H_3_O^+^ and competing proton transfer. To evaluate this effect, the ion population consisting of reactant ions H_3_O^+^, protonated acetone (ACE·H^+^) and protonated DMF (DMF·H^+^) is modeled depending on reaction time at 1000 mbar and 1.2 Td. The assumed vapor concentration of acetone is 1 ppb_V_, while two different vapor concentrations of DMF of 1 ppb_V_ and 100 ppb_V_ are considered.

Figure 2 (a) shows that for 1 ppb_V_ of both acetone (A in Figure 1) and DMF (B in Figure 1) and for short reaction times up to 0.5 ms, the relative ion concentration of ACE·H^+^ and DMF·H^+^ follows a similar trend as a function of reaction time. Under these conditions, the reaction kinetics of the initial proton transfer between H_3_O^+^ and the neutral species govern the reaction system, setting it under kinetic control. For short reaction times, the reaction rate for competing proton transfer between ACE·H^+^ and DMF is negligible due to the initially low amount of available ACE·H^+^. For short reaction times and low vapor concentrations of the neutral species, the amount of H_3_O^+^ available for initial proton transfer can be assumed constant, and thus, the reaction rate of initial proton transfer can be assumed constant. Therefore, a linear function can effectively estimate the relative ion concentration of protonated monomer AH^+^ as a function of reaction time *t*_R_, with the slope representing the product of reaction rate coefficient *k* and neutral particle density [A], as shown in eq 6. Due to charge conservation, the total ion count after reaction [I^+^]_total_ is equal to the ion count of H_3_O^+^ before reaction [H_3_O^+^]_0_. In Figure 2 (a), dashed lines denote the purely kinetic limit of product ion formation, that is valid when H_3_O^+^ is not effectively consumed and ion loss via competing proton transfer is negligible, aligning well with the modeled relative ion concentrations of both ACE·H^+^ and DMF·H^+^ for short reaction times.

$$\frac{\left[AH^{+}\right]}{{\left[I^{+}\right]}_{\mathrm{total}}}=\frac{\left[AH^{+}\right]}{{\left[H_{3}O^{+}\right]}_{0}}=k\cdot \left[A\right]\cdot t_{R}$$ 6

When reaction time increases, the modeled relative ion concentrations of ACE·H^+^ and DMF·H^+^ begin to deviate from the linear functions, as shown in Figure 2 (b). For DMF·H^+^, this deviation occurs because the reaction rate of the initial proton transfer decreases as the relative ion concentration of H_3_O^+^ decreases due to its consumption from the initial proton transfer. For ACE·H^+^, the competing proton transfer reaction rate with neutral DMF, forming DMF·H^+^, increases with longer reaction times, as the relative ion concentration of ACE·H^+^ increases. Therefore, at the longest reaction time considered (5 ms), ACE·H^+^ has only half of the relative ion concentration of DMF·H^+^. The significantly lower relative ion concentration of ACE·H^+^ compared to DMF·H^+^ at longer reaction times highlights the general challenge of accurate quantification of a target analyte in the presence of an interfering species with a higher GB.

**Figure 2:**
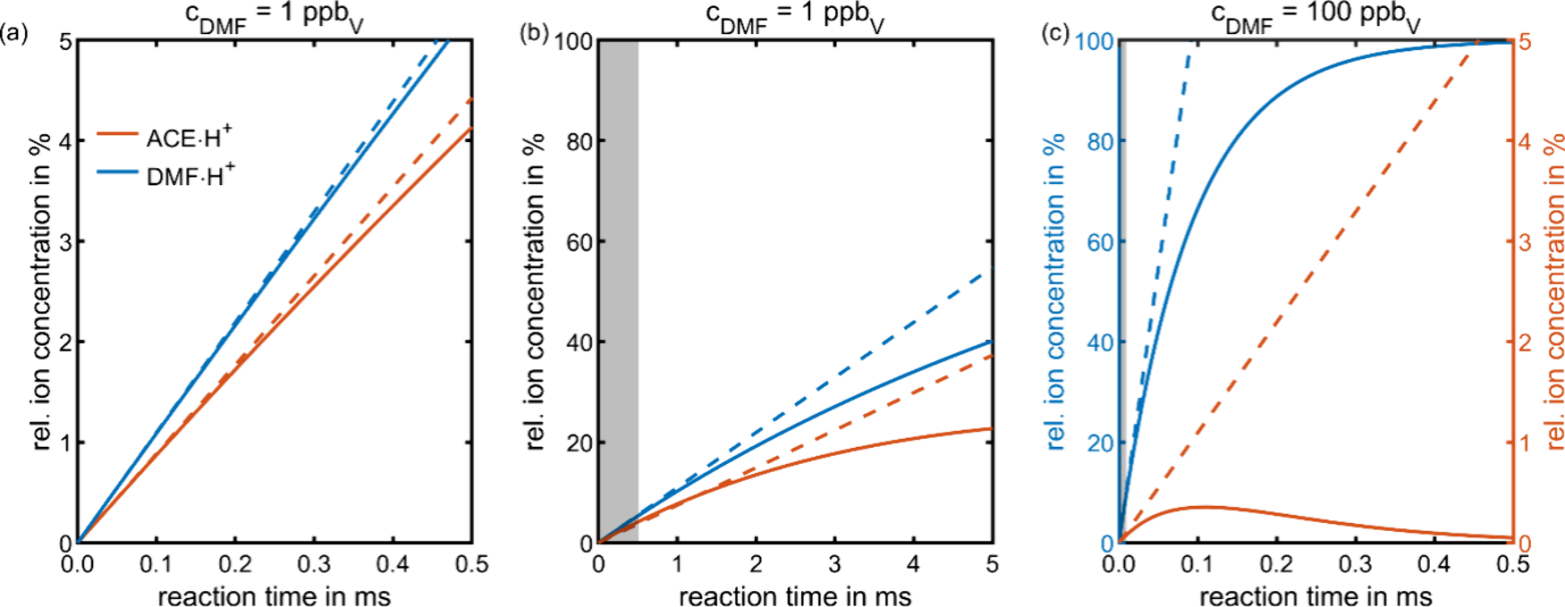
Effect of ion suppression of the protonated analyte acetone (concentration of 1 ppb_V_) by the interference DMF at 1000 mbar and 1.2 Td. Relative ion concentration of protonated acetone (ACE·H^+^) and protonated DMF (DMF·H^+^) depending on the reaction time with an interference concentration of (a) 1 ppb_V_, (b) 1 ppb_V_, and (c) 100 ppb_V_. Note the different reaction times in (a) and (b) and the different scalings in (c). The time domain in which the reaction system is kinetically controlled is highlighted in (b) and (c) by a gray area – barely visible in (c). All other parameters were set according to the standard values listed in Table 2.

The data suggest that the linear relationship from eq 6 accurately describes the relative ion concentrations of ACE·H^+^ and DMF·H^+^, meaning that the reaction system is under kinetic control, until about 10% of H_3_O^+^ are consumed. Due to charge conservation, the relative loss of H_3_O^+^ equals the sum of the relative ion concentrations of all formed product ions (here, ACE·H^+^ and DMF·H^+^) according to eq 7. Solving eq 6 for the reaction time and expressing the neutral particle density as the ratio of pressure *p* to Boltzmann constant *k*_B_ and temperature *T*, multiplied with neutral vapor concentration *c*, leads to eq 8. This equation provides an estimation for the threshold reaction time *t*_R,th_ before the reaction system starts turning to thermodynamic control. The kinetically controlled domain, according to this estimation, is highlighted in Figure 2 (b) and aligns well with the linear behavior up to the calculated threshold reaction time of 505 μs.

$$\frac{{\left[H_{3}O^{+}\right]}_{0}-{\left[H_{3}O^{+}\right]}_{t_{R}}}{{\left[H_{3}O^{+}\right]}_{0}}=\frac{\overset{n}{\underset{i=1}{\sum }}{\left[{\mathrm{AH}}^{+}\right]}_{i}}{{\left[H_{3}O^{+}\right]}_{0}}$$ 7

$$t_{R}\leq \frac{0.10}{\overset{n}{\underset{i=1}{\sum }}k_{i}\left[A_{i}\right]}=\frac{0.10}{\frac{p}{k_{B}T}\overset{n}{\underset{i=1}{\sum }}k_{i}c_{i}}=t_{R,\mathrm{th}}$$ 8

Figure 2 (b) also shows that the relative ion concentration and thus signal intensity of ACE·H^+^ increase at a longer reaction time of 5 ms compared to 0.5 ms. Although longer reaction times may not allow for accurate quantification due to the reaction system no longer being under kinetic control, they can still offer enhanced sensitivity. This indicates that the optimal operating conditions for highest sensitivity and reduced proton transfer reactions between analytes and interferents may not necessarily align, thus requiring the application to define the operating parameters.

Moreover, Figure 2 (c) reveals significant ion suppression of ACE·H^+^ at higher interference concentrations of 100 ppb_V_, starting at shorter reaction times of 50 μs due to the higher reaction rate of competing proton transfer with DMF. As the initial proton transfer between H_3_O^+^ and DMF also exhibits a higher reaction rate, the reaction system approaches thermodynamic control after 0.5 ms, with DMF being the sole ion species. This underlines that efficient ionization under such conditions is just possible for analytes with high GB. Another key finding is that shorter reaction times effectively minimize ion suppression by competing reactions regardless of interference concentration. Again, eq 8 provides an accurate estimation of the threshold reaction time of 9.0 μs before the reaction system starts turning to thermodynamic control. Therefore, when the reaction rate coefficients and expected vapor concentrations can be estimated, this relationship can help to adjust operating parameters to ensure kinetic control. Conversely, the reaction time for optimal sensitivity for analyte detection in the presence of an interfering species strongly depends on the interference concentration and therefore cannot be universally stated.

In summary, under kinetic control, both the analyte and the interfering species can be accurately quantified. However, under thermodynamic control, only the interfering species can be detected. The highest sensitivity for target analyte detection is found in the transition region between these two regimes, but accurate quantification becomes increasingly challenging or highly dependent on the interference concentration.

As discussed above, in an IMS that uses a time-independent and homogeneous electric field in the reaction region, the reaction time depends on the ion mobility of the reactant ions, the reaction region length, and the reduced electric field strength. Here, H_3_O^+^ ions ionize neutral molecules through initial proton transfer while drifting through the reaction region. Consequently, the ion population depends on the position within the reaction region, with the reaction time being a dependent variable. Hence, the ion population at the end of the reaction region is transferred into the drift region and thus analyzed by the IMS. All further calculations are based on solving the position-dependent differential equations to determine the ion populations at the end of the reaction region (here, at 50 mm from its entrance). Exemplary spatial distributions of ion populations are provided in section S3 in the Supporting Information.

Next, the impact of operating pressure on ion suppression due to competing proton transfer is analyzed. The ion population of H_3_O^+^, ACE·H^+^ and DMF·H^+^ is modeled at different operating pressures, with a constant analyte concentration of 0.1 ppm_V_ ACE and interference concentrations ranging from 1 ppb_V_ to 1 ppm_V_ DMF. To account for the lower operating pressures, the assumed analyte concentration is increased compared to previous calculations. For reference, the ion population is also modeled without any interfering species at the same analyte concentration. The reduced field strength is set to 1.2 Td for all pressures, as this is a realistic value for ambient pressure operation. In this calculation, the set of differential equations is solved depending on position in the reaction region, rather than reaction time, with the ion population at the end of the reaction region being analyzed and assumed equivalent to the detected ion population. Given equal *E*/*N* and ion mobility, the reaction time is equal across all operating pressures according to eq 5.

To better illustrate the effect of ion suppression on the protonated analyte monomer ACE·H^+^ by competing proton transfer, the relative ion concentration of ACE·H^+^ at the end of the reaction region (at 50 mm, see section S3) is normalized to its relative ion concentration from the reference calculation without interference, which is referred to as the *survival yield* in the following. A survival yield approaching 100% indicates minimal ion suppression due to competing proton transfer. Figure 3 (a) shows that, as expected, the survival yield of ACE·H^+^ decreases with increasing interference concentration due to the higher reaction rate of competing proton transfer with neutral DMF. However, for a given interference concentration, a lower operating pressure leads to a higher survival yield of ACE·H^+^, thus reducing the effect of ion suppression. This finding aligns with the expectation that lower operating pressures, while leading to lower sensitivity, minimize the effect of competing ion–molecule reactions. However, it is important to note that the sensitivity for otherwise suppressed analytes can significantly increase.

**Figure 3:**
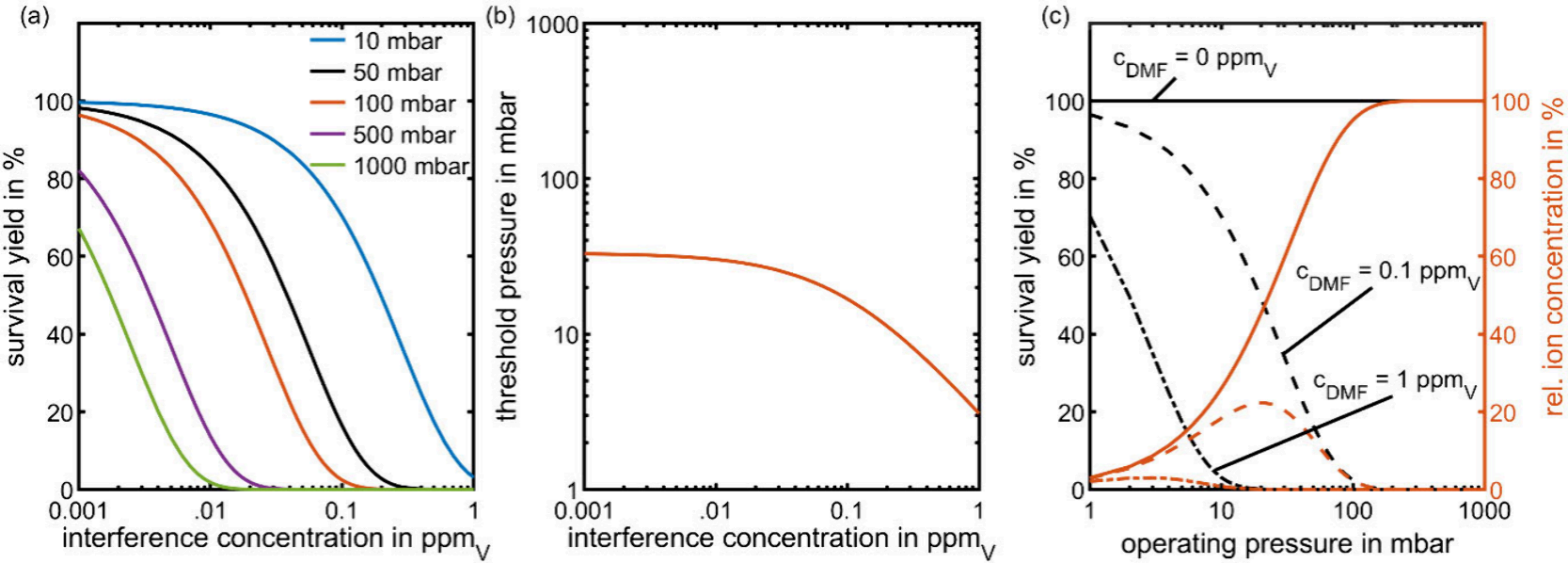
Effect of ion suppression at different operating pressures of IMS assuming a constant *E*/*N* ratio of 1.2 Td and a constant analyte concentration of 100 ppb_V_ ACE (0.1 ppm_V_). (a) Survival yield of ACE·H^+^ in the presence of DMF depending on the DMF concentration at different operating pressures. (b) Threshold operating pressure before the reaction system starts turning to thermodynamic control depending on the DMF concentration. (c) Survival yield and relative ion concentration of ACE·H^+^ in the presence of DMF depending on operating pressure for three DMF concentrations of 0, 0.1, and 1 ppm_V_. All other parameters were set according to the standard values listed in Table 2.

Similar to the definition of the threshold reaction time before the system starts turning to thermodynamic control, as defined in eq 8, it is reasonable to define a threshold operating pressure *p*_th_ before the reaction system starts turning to thermodynamic control for a given reaction time. This means that the system is under kinetic control for operating pressures below the threshold operating pressure. Its value can be estimated from eq 9, obtained by solving eq 8 for the pressure.

$$p\leq \frac{0.10}{\frac{t_{R}}{k_{B}T}\overset{n}{\underset{i=1}{\sum }}k_{i}c_{i}}=p_{\mathrm{th}}$$ 9

For an assumed analyte concentration of 0.1 ppm_V_, the threshold operating pressure before the reaction system starts turning to thermodynamic control, depending on the interference concentration, is shown in Figure 3 (b). When the interference concentration reaches a similar order of magnitude as the analyte concentration, the reaction system shifts to thermodynamic control at lower operating pressures, as the reactant ions are consumed more quickly due to the additional proton transfer with DMF. The results highlight that especially high interference concentrations necessitate low operating pressures for accurate quantification and minimizing competing reactions.

The survival yield of ACE·H^+^ characterizes ion suppression by competing proton transfer but does not indicate the total amount of formed product ions related to the sensitivity. In contrast, the relative ion concentration of an ion species within the total ion population can be used to compare the amount of product ions formed under different operating conditions, assuming a constant reactant ion generation rate independent of the operating conditions. To further elaborate on the pressure dependence of initial and competing proton transfer, both the relative ion concentration and the survival yield of ACE·H^+^ are modeled depending on operating pressures for a constant analyte concentration of 0.1 ppm_V_ ACE with three different DMF concentrations of 0, 0.1, and 1 ppm_V_ at a constant *E*/*N* of 1.2 Td. By definition, the survival yield at zero interference concentration is 100%. Figure 3 (c) shows that in the absence of DMF (solid line) the relative ion concentration of ACE·H^+^ steadily increases with increasing pressure due to the higher reaction rate for initial proton transfer with H_3_O^+^. Consequently, the reactant ions are consumed at about 100 mbar, making ACE·H^+^ the only available ion species.

When the interference concentration increases, the competing proton transfer leads to a decrease in the relative ion concentration and survival yield of ACE·H^+^ compared with the reference calculation without interference. With increasing interferent concentration, the survival yield decreases for a given operating pressure due to the higher reaction rate of competing proton transfer. In summary, these results show that for given analyte and interference concentrations, reducing the operating pressure of IMS reduces the effect of competing ion–molecule reactions, thus minimizing analyte ion suppression and allowing for accurate quantification. Furthermore, in the case of high interference concentrations, reducing the operating pressure can even lead to a higher sensitivity regarding the detection of the target analyte, as shown for an interference concentration of 0.1 ppm_V_. Without interference, choosing a higher operating pressure maximizes the sensitivity of IMS.

To consider the influence of the operating pressure on competing proton transfer, a constant *E*/*N* of 1.2 Td was assumed. However, if the operating pressure is decreased by a factor of X and the electric field is kept constant, the *E*/*N* in the reaction region is increased by the same factor X by using the same experimental setup that includes a reaction region length *L*_RR_ and a high-voltage power supply with the maximum reaction voltage *U*_RR_. According to eq 5, this also leads to a decrease in the reaction time by the same factor X (not considering any field-dependence of ion mobility). The influence of the reaction time on initial and competing proton transfer has been discussed above. Consequently, reducing the operating pressure from 1000 mbar to 10 mbar would allow for an increase in *E*/*N* from 1.2 to 120 Td. This is addressed in Figure 4 (a), which shows both the survival yield and relative ion concentration of ACE·H^+^ for a constant analyte concentration of 0.1 ppm_V_ ACE at three interference concentrations of 0, 0.1, and 1 ppm_V_ DMF. Here, it is assumed that the IMS operates at the maximum possible *E*/*N* value for each operating pressure, leading to 1.2 Td at 1000 mbar and 120 Td at 10 mbar.

**Figure 4:**
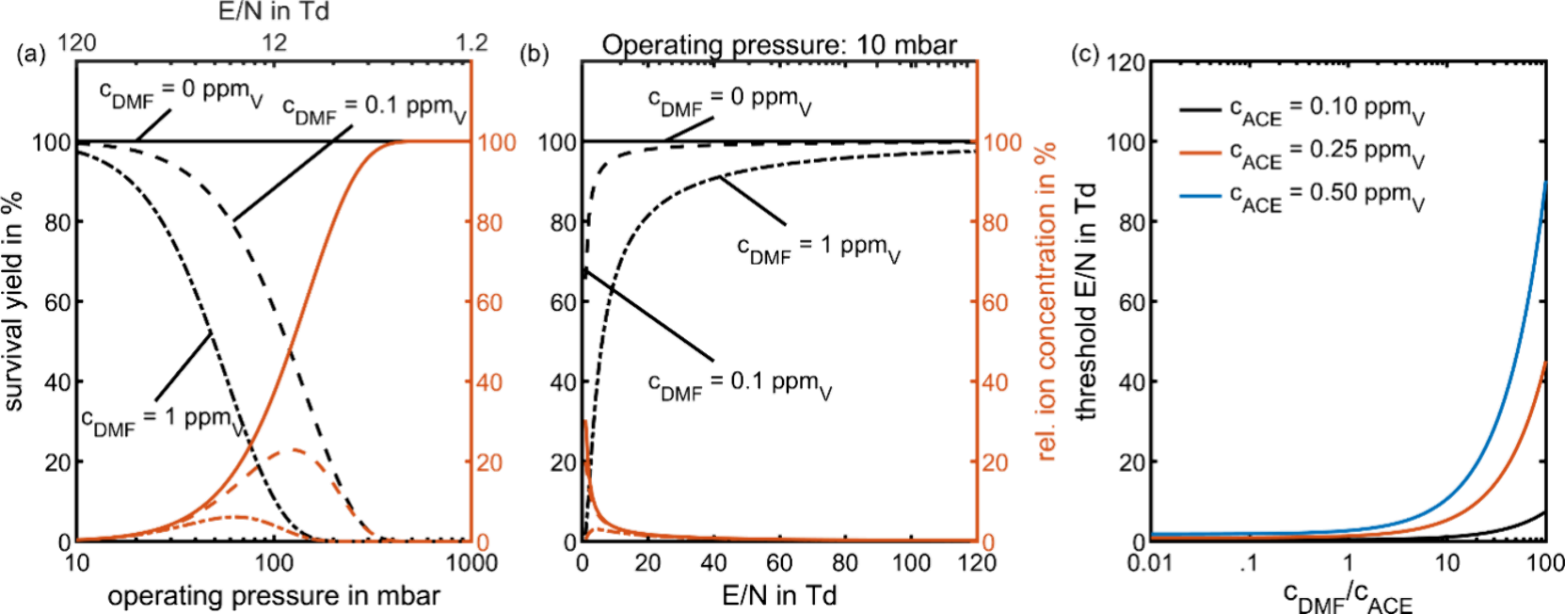
Effect of ion suppression at different reduced electric field strengths with an ACE concentration of 100 ppb_V_ (0.1 ppm_V_) for different DMF concentrations. (a) Survival yield and relative ion concentration of ACE·H^+^ depending on operating pressure. The *E*/*N* is adapted simultaneously with the operating pressure assuming a constant electric field, leading to 1.2 Td at 1000 mbar and 120 Td at 10 mbar. (b) Survival yield and relative ion concentration of ACE·H^+^ depending on *E*/*N* at a constant pressure of 10 mbar, corresponding to a change just in electric field. (c) Threshold *E*/*N*, above which kinetic control applies at a constant operating pressure of 10 mbar. All other parameters were set according to the standard values listed in Table 2.

Comparing the survival yields in Figure 4 (a) and Figure 3 reveals that the increase in *E*/*N* at low operating pressures leads to even higher survival yields for ACE·H^+^, effectively reducing the impact of competing proton transfer. Figure 4 demonstrates a notable difference in survival yield between operating at low pressure (*i.e*. high *E*/*N*) and operating at high pressure (i.e., low *E*/*N*), as ACE·H^+^ has a significantly higher survival yield, and for high interferent concentrations also higher sensitivity, at high *E*/*N* compared to low *E*/*N*. Thus, increasing the *E*/*N* enhances the sensitivity and reduces the number of proton transfer reactions between analytes and interferents. Here, both the lower ion–neutral collision frequency at reduced pressures and shorter reaction times at higher *E*/*N* contribute to minimizing the competing proton transfer, thereby favoring kinetic control.

Operating the IMS at low pressure allows *E*/*N* to vary rapidly over a wide range simply by adjusting the reaction voltage at a constant pressure. This control of the reaction time allows for choosing between enhancing sensitivity (with the longest reaction time corresponding to low *E*/*N*) and reducing proton transfer reactions between analytes and interferents (with the shortest reaction time corresponding to high *E*/*N*). To investigate this effect, the relative ion concentration and survival yield of ACE·H^+^ are modeled with an analyte concentration of 0.1 ppm_V_ at a constant pressure of 10 mbar, while varying *E*/*N* between 1 and 120 Td by varying the electric field strength for three different DMF concentrations of 0 ppm_V_, 0.1 ppm_V_, and 1 ppm_V_. Figure 4 (b) shows that, as expected, in the absence of DMF, ACE·H^+^ achieves the highest relative ion concentration at 1 Td, which declines at higher *E*/*N* due to the reduced reaction time. Conversely, at higher interference concentrations, the maximum relative ion concentration of ACE·H^+^ occurs at higher *E*/*N* values, since the effect of competing proton transfer leading to loss of ACE·H^+^ is most pronounced at the longest reaction times and lowest *E*/*N*. While the survival yield steadily increases with higher *E*/*N*, ensuring accurate quantification at high *E*/*N*, the relative ion concentration drops after achieving a maximum value at 4 Td for 1 ppm_V_ of DMF. This demonstrates that varying *E*/*N* can quickly switch between operating points for optimal sensitivity and a minimum number of proton transfer reactions between analytes and interferents. It is important to note that *E*/*N* can also influence the reaction rate coefficient for bimolecular reactions, such as the initial proton transfer with H_3_O^+^, and may cause fragmentation of product ions.^76–79^ These changes to the ion population are not accounted for in this work but need to be considered when varying the *E*/*N*.

The threshold reduced field strength (*E*/*N*)_th_ above which the reaction system is under kinetic control for a given operating pressure can be estimated from eq 10, that is obtained by solving eq 8 for the reduced field strength, given the reaction time from eq 5. The obtained threshold values of *E*/*N* in Figure 4 (c) show that especially for higher analyte and interference concentrations, kinetic control is only achieved at high *E*/*N*, confirming the results from Figure 4 (b).

$$\frac{E}{N}\geq \frac{L_{\mathrm{RR}}\cdot \frac{p}{k_{B}T}\cdot \overset{n}{\underset{i=1}{\sum }}k_{i}c_{i}}{0.10\cdot K_{0}\cdot N_{0}}={\left(\frac{E}{N}\right)}_{\mathrm{th}}$$ 10

## Conclusions

This work investigated the ion–molecule reactions occurring in IMS including the effect of competing reactions by applying kinetic modeling. The results clearly demonstrate that the reaction time, reaction region length, operating pressure, and *E*/*N* are effective parameters for controlling the reaction system, ensuring either kinetic or thermodynamic control, as they all affect the number of ion–neutral collisions. Often, the reaction region length and operating pressure are determined by experimental or instrumental constraints such as the high-voltage power supply, vacuum pumps, achievable *E*/*N*, or the limited size of mobile or hand-held instruments. In contrast, *E*/*N* and reaction time can be adjusted quickly over a wide range. However, *E*/*N* can significantly impact ion chemistry.^76–79^ Additionally, in IMS that use a homogeneous, time-independent electric field in the reaction region, reaction time is determined by the reaction region length, ion mobility of reactant ions, and *E*/*N*. Modifications to the IMS reaction region that would allow for freely adjusting reaction times while ionizing in an electric field could address these limitations. Nevertheless, the reaction time remains the most promising parameter for controlling the reaction system to ensure optimal sensitivity and minimizing ion suppression from competing ion–molecule reactions. The remaining operating parameters (pressure, *E*/*N*, reaction region length) can then be adjusted to preselect the feasible operating range. However, focusing only on one parameter is insufficient; reaction time, pressure and field strength must all be evaluated together to steer ion chemistry toward optimum sensitivity and minimal competing ion–molecule reactions.

Particularly, operating at low pressure and high reduced electric field strengths ensures kinetic control of ion formation, allowing for a minimum number of proton transfer reactions between analytes and interferents and thus accurate quantification. Such conditions enable the detection of analytes with a low gas basicity in complex chemical backgrounds. Since lower operating pressures also lead to decreased reaction rates of initial proton transfer by the reactant ions, a compromise for detecting analytes with low gas basicity needs to be found to ensure sufficient ionization while still reducing proton transfer reactions between analytes and interferents. In contrast, elevated operating pressures or longer reaction times lead the reaction system to chemical equilibrium, favoring ionization based on suitable thermodynamic properties, such as high gas basicity. While this discriminates certain analytes in complex chemical backgrounds, it ensures efficient ionization of target analytes in less complex backgrounds as well as target analytes having high gas basicity, even in complex backgrounds. Hence, for detecting target analytes with high gas basicity, operation at ambient pressure maximizes sensitivity since further competing reactions are unlikely for these analytes. Note that this work did not include a reverse reaction for proton transfer, which is valid given the large differences in gas basicity among the considered species. However, in cases of similar gas basicities, such reactions can also substantially affect ion chemistry. While this work focused on proton transfer, the findings can also be applied to other bimolecular ion–molecule reactions such as charge transfer with NO^+^ or O_2_^+•^. Moreover, similar considerations could be extended to MS using chemical ionization if the relevant parameters are varied over a wide range.

## Supplementary Material





## Notes

Detailed description of the kinetic model; a derivation of the equation for calculating the effective temperature from the Wannier equation; discussion on influence of reaction region (PDF)
Data on effective temperature and reaction rate coefficients for all three reactions depending on *E*/*N* (XLSX)
