# Supplementary material for: Sensitivity vs Competing Proton Transfer Reactions: Addressing Key Parameters of Ion Chemistry in Ion Mobility Spectrometry
Source: J Am Soc Mass Spectrom. 2025 Aug 1;36(9):1929–39. doi: 10.1021/jasms.5c00161 (PMC12412161; doi:10.1021/jasms.5c00161)
Supplement: Supplementary file 1 [file js5c00161_si_001.pdf]

Supporting Information

Sensitivity vs. Competing Proton Transfer Reactions: Addressing Key Parameters on Ion Chemistry in Ion Mobility Spectrometry

Christoph Schaefer*, Stefan Zimmermann

Leibniz University Hannover, Institute of Electrical Engineering and Measurement Technology, Department of Sensors and Measurement Technology, Appelstr. 9A, 30167 Hannover, Germany

*Corresponding Authors: schaefer@geml.uni-hannover.de;

Content

S1. Kinetic model S-2

S2. Derivation of the effective temperature from Wannier's equation S-5

S3. Position-dependent ion populations in the reaction region of IMS S-6

References S-7

S1. Kinetic model

The kinetic model considers reactions between an initial reactant ion species H_3O^+ with each considered neutral molecule within the reaction region, as well as all competing ion-molecule reactions between the formed product ions and all present neutral molecules. In this work, this includes the ionization of the analyte acetone (ACE) and interfering species dimethylformamide (DMF) by initial proton transfer as well as the competing proton transfer between them given by the reactions (1),(2) and (3). Typically, it is assumed that these proton transfer reactions occur at each collision, if the reaction is exothermic by more than 25 kJ/mol.[1] This condition is met for water, ACE, and DMF with GB(H_2O) = 660.0 kJ/mol, GB(ACE) = 782.1 kJ/mol and GB(DMF) = 856.6 kJ/mol.[2]

$$\mathrm{H_3O^+ + ACE \rightarrow ACE \cdot H^+ + H_2O} \tag{1}$$

$$\mathrm{H_3O^+ + DMF \rightarrow DMF \cdot H^+ + H_2O} \tag{2}$$

$$\mathrm{ACE \cdot H^+ + DMF \rightarrow DMF \cdot H^+ + ACE} \tag{3}$$

Common practice in SIFT-MS and PTR-MS,[3,4] relying on the same ion-molecule reaction as IMS, is to calculate the reaction rate coefficients from the collision rates given by the parametrization from Su and Chesnavich from equation (4).[3] Only considering the Langevin rate coefficient k_L from equation (5), depending on the ion charge q, the neutral polarizability α and the reduced mass μ would only consider the repulsive interaction potential as well as an ion-induced dipole interaction. To also include the ion-permanent dipole interaction, the Langevin rate coefficient is multiplied with the capture coefficient according to equation (4) to yield the capture rate according to Su and Chesnavich. The empirically determined estimation of the capture coefficient is given by equations (6), (7) and (8), where μ_D is the dipole moment of the neutral particle. The calculation relies on the effective temperature, which describes the mean relative energy in collisions and depends on the ambient temperature T, the Boltzmann constant k_B, the reduced ion mobility K_0, the Loschmidt constant N_0 and the reduced field strength E/N. Hence, when varying the reduced field strength, the capture rate needs to be calculated for each value of effective temperature. A derivation of equation (8) from the work of Wannier[5] can be found in the following section *S2*. The capture rates depending on E/N, being assumed equal to the reaction rate coefficients for the reactions (1),(2) and (3) can be found in the attached .xlsx file.

$$k_\mathrm{cap}(T_\mathrm{eff}) = k_\mathrm{L} \cdot K_\mathrm{cap} \tag{4}$$

$$k_{\mathrm{L}} = 2\pi q \sqrt{\frac{\alpha}{\mu}} \tag{5}$$

$$K_{\mathrm{cap}} = \begin{cases} 0.4767 \cdot x + 0.6200 \\ \frac{(x + 0.5090)^2}{10.526} + 0.9754 \end{cases} \tag{6}$$

$$x = \frac{\mu_{\mathrm{D}}}{\sqrt{2\alpha k_{\mathrm{B}} T_{\mathrm{eff}}}} \tag{7}$$

$$T_{\mathrm{eff}} = T + \frac{M}{3k_{\mathrm{B}}}\left(K_0 N_0 \frac{E}{N}\right)^2 \tag{8}$$

The change in ion concentration for each species can be calculated from the reaction rate coefficient for each reaction multiplied by the number densities of the involved ion and neutral species. The neutral number densities are calculated by multiplying the neutral concentration (more specifically, its volume fraction) ϕ_M) with the total neutral number density, which is given as the ratio of pressure p and the product of Boltzmann constant and temperature assuming an ideal gas as shown in eq. (12). In case of the ion species, it is convenient to calculate with relative ion concentrations (see below). This leads to the system of three differential equations according to equations (9), (10) and (11). Solving these differential equations over time provides the time-dependent solution for the ion population.

$$\frac{\mathrm{d}[\mathrm{H_3O^+}]}{\mathrm{d}t} = -k_{\mathrm{for,1}}[\mathrm{H_3O^+}][\mathrm{ACE}] - k_{\mathrm{for,2}}[\mathrm{H_3O^+}][\mathrm{DMF}] \tag{9}$$

$$\frac{\mathrm{d}[\mathrm{ACE \cdot H^+}]}{\mathrm{d}t} = k_{\mathrm{for,1}}[\mathrm{H_3O^+}][\mathrm{ACE}] - k_{\mathrm{for,3}}[\mathrm{ACE \cdot H^+}][\mathrm{DMF}] \tag{10}$$

$$\frac{\mathrm{d}[\mathrm{DMF \cdot H^+}]}{\mathrm{d}t} = k_{\mathrm{for,2}}[\mathrm{H_3O^+}][\mathrm{DMF}] + k_{\mathrm{for,3}}[\mathrm{ACE \cdot H^+}][\mathrm{DMF}] \tag{11}$$

$$[\mathrm{M}] = \phi_M \frac{p}{k_{\mathrm{B}} T} \tag{12}$$

In a reaction region of an IMS with applied electric field, the ions are continuously transported through the reaction region. Usually, the reaction system is interrupted at the end of the reaction region. In the instruments, this is realized by ensuring that the sample gas is only present in the reaction region. Hence, in such IMS, the ion population will change when being transported through the reaction region, but it will be constant over time at a given position assuming that the other instrumental parameters remain constant. To

describe the position-dependent ion population, the time-dependent set of differential equations is divided by the drift velocity of the drifting ion species according to equation (13). The reduced ion mobilities required for calculating the drift velocity as in equation (14) for each ion species and every *E*/*N* value, the field-dependent ion mobilities are calculated with MobCal-MPI using the routine explained in the main manuscript. Equation (14) highlights that the electric field, or more specifically, the reduced electric field strength, mainly affects the model results due to its influence on the drift velocity on thus on reaction times. However, as it also affects the reaction rate coefficients, its influence is rather complex.

$$\frac{\mathrm{d}[\mathrm{I}^+]}{\mathrm{d}x} = \frac{\mathrm{d}[\mathrm{I}^+]}{\mathrm{d}t} \cdot \frac{1}{v_{D,I^+}} \quad (13)$$

$$v_{D,I^+} = K_{0,I^+} N_0 \frac{E}{N} \quad (14)$$

The assumed low-field reduced ion mobilities for H_3O^+, $ACE \cdot H^+$ and $DMF \cdot H^+$, calculated with MobCal-MPI, compared to a simple estimation of the ion mobility from the ion mass as implemented in the statistical diffusion simulation (SDS) user program for SIMION[6] and experimental values from the literature[7–9] are summarized in Table 1. While the simple estimation of the ion mobility from the SDS model deviates from the values calculated for H_3O^+, it provides a good agreement for the larger ions $ACE \cdot H^+$ and $DMF \cdot H^+$. At low *E*/*N*, where the low-field ion mobility is determined, these ion species readily form hydrates so that the experimental values under such conditions are lower than the values from MobCal-MPI, since they do not reflect the reduced ion mobility of the bare ion. In contrast, at high *E*/*N*, where the hydrates dissociate, other field-dependent effects such as the hard-sphere effect and the influence of relative ion-neutral velocity on their interaction potential affect the ion mobilities, again leading to differences from the low-field ion mobility. To the best of our knowledge, no ion mobility values have been reported for DMF. Therefore, we decided to apply the low-field ion mobilities from MobCal-MPI in this work.

Table 1. Comparison between the low-field reduced ion mobility calculated with MobCal-MPI[10] and ion mobilities obtained with the simplified estimation from the SDS model[6] and experimental values (exp.) in the low-field limit and at high E/N of 120 Td.

Ion species	K_0 (MobCal-MPI) in cm^2/Vs	K_0 (SDS model) in cm^2/Vs	K_0 (exp. at low-field limit) in cm^2/Vs	K_0 (exp. at 120 Td) in cm^2/Vs
H_3O^+	2.7489	3.5663	2.06[7]	2.891[9]
$ACE \cdot H^+$	2.2440	2.2800	1.99[7]	2.36[8]
$DMF \cdot H^+$	2.0758	2.0859	N/A	N/A

It is convenient to conduct all calculations using relative ion concentrations, meaning that the sum of all ion concentrations equals 1. Assuming that only H_3O^+ is present initially leads to a relative ion concentration of 1 for H_3O^+ and a relative concentration of 0 for both ACE·H$^+$ and DMF·H$^+$ in the first time step or at the beginning of the reaction region (see equation (15)). This leads to the sets of differential equations in equations (16) and (17).

$$\begin{pmatrix} [\mathrm{H_3O^+}] \\ [\mathrm{ACE}\cdot\mathrm{H^+}] \\ [\mathrm{DMF}\cdot\mathrm{H^+}] \end{pmatrix}_{t=0} = \begin{pmatrix} 1 \\ 0 \\ 0 \end{pmatrix}; \begin{pmatrix} [\mathrm{H_3O^+}] \\ [\mathrm{ACE}\cdot\mathrm{H^+}] \\ [\mathrm{DMF}\cdot\mathrm{H^+}] \end{pmatrix}_{x=0} = \begin{pmatrix} 1 \\ 0 \\ 0 \end{pmatrix} \tag{15}$$

$$\begin{pmatrix} \frac{\mathrm{d}[\mathrm{H_3O^+}]}{\mathrm{d}t} \\ \frac{\mathrm{d}[\mathrm{ACE}\cdot\mathrm{H^+}]}{\mathrm{d}t} \\ \frac{\mathrm{d}[\mathrm{DMF}\cdot\mathrm{H^+}]}{\mathrm{d}t} \end{pmatrix} = \begin{pmatrix} -k_{\mathrm{for},1}[\mathrm{ACE}] - k_{\mathrm{for},2}[\mathrm{DMF}] & 0 & 0 \\ k_{\mathrm{for},1}[\mathrm{ACE}] & -k_{\mathrm{for},3}[\mathrm{DMF}] & 0 \\ k_{\mathrm{for},2}[\mathrm{DMF}] & k_{\mathrm{for},3}[\mathrm{DMF}] & 0 \end{pmatrix} \begin{pmatrix} [\mathrm{H_3O^+}] \\ [\mathrm{ACE}\cdot\mathrm{H^+}] \\ [\mathrm{DMF}\cdot\mathrm{H^+}] \end{pmatrix} \tag{16}$$

$$\begin{pmatrix} \frac{\mathrm{d}[\mathrm{H_3O^+}]}{\mathrm{d}x} \\ \frac{\mathrm{d}[\mathrm{ACE}\cdot\mathrm{H^+}]}{\mathrm{d}x} \\ \frac{\mathrm{d}[\mathrm{DMF}\cdot\mathrm{H^+}]}{\mathrm{d}x} \end{pmatrix} = \begin{pmatrix} \frac{1}{v_{D,H_3O^+}}(-k_{\mathrm{for},1}[\mathrm{ACE}] - k_{\mathrm{for},2}[\mathrm{DMF}]) & 0 & 0 \\ \frac{1}{v_{D,H_3O^+}} k_{\mathrm{for},1}[\mathrm{ACE}] & \frac{1}{v_{D,FA\cdot H^+}} - k_{\mathrm{for},3}[\mathrm{DMF}] & 0 \\ \frac{1}{v_{D,H_3O^+}} k_{\mathrm{for},2}[\mathrm{DMF}] & \frac{1}{v_{D,FA\cdot H^+}} k_{\mathrm{for},3}[\mathrm{DMF}] & 0 \end{pmatrix} \begin{pmatrix} [\mathrm{H_3O^+}] \\ [\mathrm{ACE}\cdot\mathrm{H^+}] \\ [\mathrm{DMF}\cdot\mathrm{H^+}] \end{pmatrix} \tag{17}$$

This system of differential equations is solved in each time step using the MATLAB function "ode15s", which employs numerical differentiation formulas that are linear multistep methods of order 1 through 5. Thus, in each time step, the relative ion concentration vector is used as input to calculate the subsequent relative ion concentration vector. In IMS, the ion population that is present at the end of the reaction region is introduced into the drift region and thus analyzed by the IMS. Therefore, in the parts discussing the influence of operating pressure and reduced electric field strength in the main manuscript, the ion population is solved depending on the position in the reaction region and the resulting ion population at the end of the reaction region (here, with a assumed length of 50 mm) is further analyzed.

S2. Derivation of the effective temperature from Wannier's equation

The derivation of the equation of the effective temperature follows the work of Mason and McDaniel.[11] The mean relative energy of collisions $\bar{\epsilon}$ is equal to the product of effective temperature and $3k_\mathrm{B}/2$ as depicted in equation (19). In this equation, μ is the reduced mass

and $\overline{v_r^2}$ is the mean relative ion-neutral velocity. The relative velocity is given by equation (19), considering that the term $\overline{v_i v_N}$ averages to zero.

$$\overline{v_r^2} = \overline{(v_i - v_N)^2} = \overline{v_i^2} + \overline{v_N^2} \tag{18}$$

$$\bar{\epsilon} = \frac{3}{2}k_{\mathrm{B}}T_{\mathrm{eff}} = \frac{1}{2}\mu\overline{v_r^2} = \frac{1}{2}\frac{m_i M_N}{m_i + M_N}\left(\overline{v_i^2} + \overline{v_N^2}\right) \tag{19}$$

Ultimately, the effective temperature can be derived from equation (20) as given by the work of Wannier.[5] Here, m_i and M_N are the masses of the ion and neutral molecule, $\overline{v_i^2}$ is the mean ion velocity, $\overline{v_N^2}$ the mean neutral velocity and $\overline{v_D^2}$ the mean drift velocity. Thus, the first term describes the thermal energy from collisions with the gas (and is thus equal to $3k_{\mathrm{B}}T/2$). The second term is the energy acquired from the electric field and visible as drift motion, while the last term corresponds to the part of the field energy that is randomized through collisions.

Rearranging equation (19) and equating with equation (20) gives equation (21). Further rearrangement finally leads to the expression for the mean relative energy of collisions in equation (22). As discussed above, the mean relative energy of collisions is equal to the product of effective temperature and $3k_{\mathrm{B}}/2$ and the first term on the right side is equal to $3k_{\mathrm{B}}T/2$ as shown in equation (23). Simple rearrangement for the effective temperature leads to the equation for calculating the effective temperature from equation (8).

$$\frac{m_i\overline{v_i^2}}{2} = \frac{M_N\overline{v_N^2}}{2} + \frac{m_i\overline{v_D^2}}{2} + \frac{M_N\overline{v_D^2}}{2} \tag{20}$$

$$\frac{m_i\overline{v_i^2}}{2} = \frac{m_i\overline{v_r^2}}{2} - \frac{m_i\overline{v_N^2}}{2} = \frac{M_N\overline{v_N^2}}{2} + \frac{m_i\overline{v_D^2}}{2} + \frac{M_N\overline{v_D^2}}{2} \tag{21}$$

$$\frac{\mu\overline{v_r^2}}{2} = \frac{M_N\overline{v_N^2}}{2} + \frac{M_N\overline{v_D^2}}{2} \tag{22}$$

$$\frac{3}{2}k_B T_{eff} = \frac{3}{2}k_B T + \frac{M_N\overline{v_D^2}}{2} \tag{23}$$

S3. Position-dependent ion populations in the reaction region of IMS

Exemplary ion populations based on position within the reaction region are shown in Figure 1 at 1000 mbar and an analyte concentration of 1 $\mathrm{ppb_V}$ ACE, both without the interfering

species DMF (Figure 1 (a)) and with an interference concentration of 1 ppb_V DMF (Figure 1*Figure 1* (b)).

The data clearly show that as ions move through the reaction region, reactant ions are consumed while $ACE \cdot H^+$ and $DMF \cdot H^+$ are formed through initial proton transfer. In Figure 1 (b), the impact of competing proton transfer is evident. Notably, increasing the reaction region length while maintaining constant ion mobility and *E*/*N* has the same effect on initial and competing proton transfer, and thus on sensitivity and proton transfer reactions between analytes and interferents, as increasing the reaction time. Consequently, a detailed discussion of the effect of reaction region length is not necessary. However, it is important to note that the shown dependence of ion population on the position within the reaction region remains constant over time.

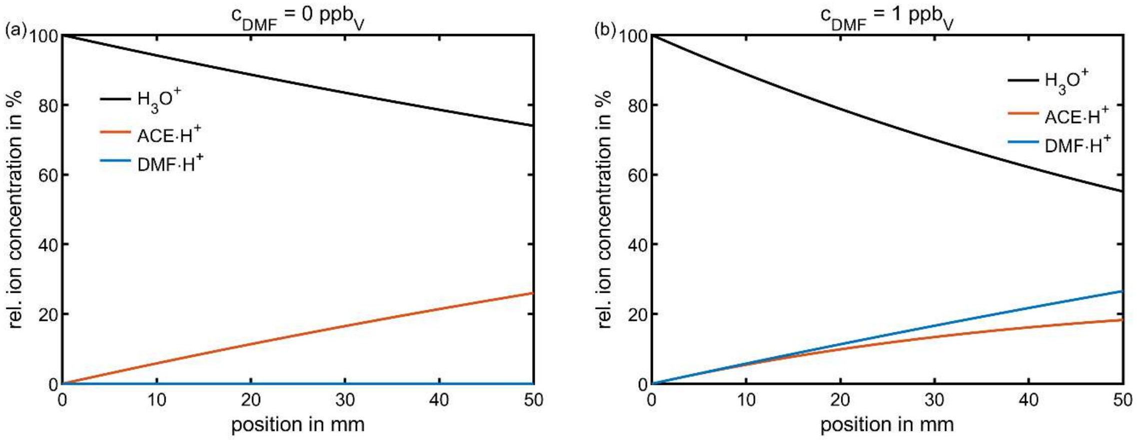

Figure 1. Relative ion concentration *of H_3O^+, $ACE \cdot H^+$, and $DMF \cdot H^+$ depending on the position in the reaction region at 1000 mbar and 1.2 Td, with an analyte concentration of 1 ppb_V ACE and an interference concentration of (a) 0 ppb_V DMF and (b) 1 ppb_V DMF. All other parameters were set according to the standard values listed in Table 3 in the main manuscript.*

References

(1) Bouchoux, G.; Salpin, J. Y.; Leblanc, D. A Relationship between the Kinetics and Thermochemistry of Proton Transfer Reactions in the Gas Phase. *Int. J. Mass Spectrom. Ion Processes* **1996**, *153* (1), 37–48. https://doi.org/10.1016/0168-1176(95)04353-5.

(2) Hunter, E. P. L.; Lias, S. G. Evaluated Gas Phase Basicities and Proton Affinities of Molecules: An Update. *J. Phys. Chem. Ref. Data* **1998**, *27* (3), 413–656. https://doi.org/10.1063/1.556018.

(3) Langford, V. S.; Dryahina, K.; Španěl, P. Robust Automated SIFT-MS Quantitation of Volatile Compounds in Air Using a Multicomponent Gas Standard. *J. Am. Soc. Mass Spectrom.* **2023**, *34* (12), 2630–2645. https://doi.org/10.1021/jasms.3c00312.

(4) Sekimoto, K.; Li, S.-M.; Yuan, B.; Koss, A.; Coggon, M.; Warneke, C.; de Gouw, J. Calculation of the Sensitivity of Proton-Transfer-Reaction Mass Spectrometry (PTR-MS) for Organic Trace Gases Using Molecular Properties. *Int. J. Mass Spectrom.* **2017**, *421*, 71–94. https://doi.org/10.1016/j.ijms.2017.04.006.

(5) Wannier, G. H. Motion of Gaseous Ions in Strong Electric Fields. *Bell Labs Tech. J.* **1953**, *32* (1), 170–254.

(6) Appelhans, A. D.; Dahl, D. A. SIMION Ion Optics Simulations at Atmospheric Pressure. *Int. J. Mass Spectrom.* **2005**, *244* (1), 1–14. https://doi.org/10.1016/j.ijms.2005.03.010.

(7) Vautz, W.; Schwarz, L.; Hariharan, C.; Schilling, M. Ion Characterisation by Comparison of Ion Mobility Spectrometry and Mass Spectrometry Data. *Int. J. Ion Mobil. Spectrom.* **2010**, *13* (3–4), 121–129. https://doi.org/10.1007/s12127-010-0051-8.

(8) Schaefer, C.; Kirk, A. T.; Allers, M.; Zimmermann, S. Ion Mobility Shift of Isotopologues in a High Kinetic Energy Ion Mobility Spectrometer (HiKE-IMS) at Elevated Effective Temperatures. *J. Am. Soc. Mass Spectrom.* **2020**, *31* (10), 2093–2101. https://doi.org/10.1021/jasms.0c00220.

(9) Allers, M.; Kirk, A. T.; Schaefer, C.; Erdogdu, D.; Wissdorf, W.; Benter, T.; Zimmermann, S. Field-Dependent Reduced Ion Mobilities of Positive and Negative Ions in Air and Nitrogen in High Kinetic Energy Ion Mobility Spectrometry (HiKE-IMS). *J. Am. Soc. Mass Spectrom.* **2020**, *31* (10), 2191–2201. https://doi.org/10.1021/jasms.0c00280.

(10) Haack, A.; Ieritano, C.; Hopkins, W. S. MobCal-MPI 2.0: An Accurate and Parallelized Package for Calculating Field-Dependent Collision Cross Sections and Ion Mobilities. *Analyst* **2023**, *148* (14), 3257–3273. https://doi.org/10.1039/D3AN00545C.

(11) Mason, E. A.; McDaniel, E. W. *Transport Properties of Ions in Gases*; Wiley, 1988. https://doi.org/10.1002/3527602852.
